# Supplementary material for: Are carbon emissions trading and green financial instruments synergistic? -Comprehensive quantitative research based on content analysis
Source: PLoS One. 2024 Mar 7;19(3):e0298601. doi: 10.1371/journal.pone.0298601 (PMC10919601; doi:10.1371/journal.pone.0298601)
Supplement: S1 Data — (ZIP) [file pone.0298601.s001.zip › Data/地级市Z_F工作报告.zip!/║■▒▒/╗╞╕╘╩╨/└┤╫╘╣1⁄2╓┌║┼í╛┬φ┐╦╩2╛▌═°í┐_2005.docx]

各位代表：

　　现在，我代表市人民政府向大会作政府工作报告，请予审议，并请市政协委员和各位列席人员提出意见。

　　一、2004年工作回顾

　　2004年，在市委正确领导下，市政府团结带领全市人民，抢抓机遇，加快发展，较好地完成了市二届人大三次会议确定的各项任务。

　　——突出第一要务，促进市域经济全面增长。坚持“全党抓经济，重点抓工业，突出抓招商 ，着力抓环境”，市域经济发展速度加快、结构改善、效益提高、活力增强。全市完成生产总值432.5亿元,比上年增长9.8%,是1999年以来的最高增幅。其它各项主要经济指标，基本上都是进入新世纪以来完成年初预期目标最好、实现增长幅度最高的一年。农业突出龙头企业、板块经济、粮食生产三个重点，在政策调动、价格拉动、行政推动和有利气候条件等综合因素的共同作用下，掀起了家庭联产承包后的又一个高潮，全市农业增加值达到108.5亿元，增长11.3%。大力推进新型工业化，加大工业园区和支柱产业、骨干企业的建设力度，工业 产销和质效同步上升。全市完成工业增加值162.6亿元，增长9.6%。其中规模以上工业增加 值68亿元，增长16.2%，利润和利税分别增长26.8% 和20.7%，工业经济效益综合指数达到131.7%。加快发展第三产业，积极扩大内需和外贸出口，全市第三产业实现增加值129.5亿元,增长9.1%;社会商品零售总额151.2亿元，增长10.7%;金融机构年末存贷款余额 分别 达到310.8亿元和205.5亿元，比年初增长21%和9.1%;外贸出口1.3亿美元，增长26.6 %;旅 游、教育、保险、房地产、现代通信、社区服务等新兴产业保持较快的发展势头。全市实现 财政收入26亿元，比上年增长16.7%，其中地方一般预算收入13.2亿元，增长20.7%。

　　——深入推进各项改革，从体制上为经济社会发展注入新的活力。以落实中央和省支 农惠农 政策为契机，整体推进农村税费改革、乡镇综合配套改革、农村教育管理体制改革、国有农 场管理体制改革、粮食流通体制改革以及农村卫生体制、血防体制、动物疫病防治体制等改 革，积极化解村级债务，农村发展活力明显增强，农民负担明显减轻。全市发放粮食直补和良种补贴过亿元;农民税费负担总额减少1.6亿元，人平负担减少30.82元;减少乡镇财政供 养人员1214人，每年可减支1100多万元。通过转公、退养、辞退等“三个一批”的办法，整 体解决了全市9130名民办教师的问题。国有企业改革继续推进，全市90%以上的国有和乡镇 集体企业走向了民营化，市属工业企业已全部改制为民有民营，市级国有资产监管体制基本 建立。政府机构改革、行政审批制度改革、财政管理体制改革、出口退税机制改革、投资管理体制改革、国土资源管理体制改革、事业单位改革等都取得了新的进展。

　　——加大以招商引资为重点的项目建设力度，增强经济和社会发展后劲。认真贯彻国家宏观调控措施，把向上争取投资和对外招商引资有机结合起来，大力支持符合国家产业政策的行业、企业、产品和项目，促进了固定资产投资的大幅增长。全社会固定资产投资完成130亿元，比上年增长22%。一期动态投资49.6亿元的大别山火电厂、总投资38.8亿元的白莲河抽水蓄能电站开工建设，黄冈天然气主管道穿江工程地质勘探全面完成，大型优质商品粮生产基地建设项目落实资金5000万元，大别山红色旅游开发项目列入国家和省投资计划、已安排 红安革命传统教育基地专项投资7000万元，江北一级公路累计完成投资6.2亿元、已基本建成和试通车，穿越我市95.5公里、境内投资达57亿元的沪汉蓉铁路快速通道已经通过评审。招商引资力度进一步加大，赴国外、境外和省外招商活动成效明显。全市引进及签约项目1236个,项目总投资236.9亿元，实际到位资金45.8亿元，其中新引进“三资”企业37家，实际 到位外资5707万美元;总投资亿元以上项目23个，新老招商引资项目当年到位资金59.7亿元 。积极参与武汉城市圈建设，制订了黄冈融入武汉城市圈建设实施意见，衔接了一批基础设施配套和产业合作项目。

　　——加速城镇建设和发展，进一步提升黄冈城市形象。坚持抓市区带全市，一手高昂市区龙 头，一手激活县域经济。市区主要经济指标完成情况好于上年，好于全市平均水平，市区十件实事大多完成或超额完成了年度工作任务，成功举办了第四届东坡赤壁文化旅游节暨黄冈中学百年校庆。城乡规划、建设、管理水平进一步提高。市区投入城建资金1.58亿元，新建 和配套了一批促进投资创业和改善人民生活的市政设施。实施“一把手”工程，建立健全长效机制，全方位、高标准、持续性强化“门前四包”，取缔麻木和摩托车非法载客营运，实 施城市交通“三让一整治”，城市形象进一步提升。各县市区累计完成城建投资7.5亿元， 加快了县市城区、中心镇、口子镇和中心村建设步伐。

　　——坚持以人为本，着力解决事关人民群众切身利益的问题。全市新增就业岗位3.95 万个, 安置下岗失业人员2.58万人;筹集小额贷款担保基金1000万元，发放优惠证3.7万本，为2.4 5万名下岗人员减免税收和各类行政事业性收费2331万元。全市各类社会保险扩面5.5万人，接续保险关系1.05万人，清欠社会保险费1950万元，保障了“两金一费”按时足额发放。全 市进入低保人数达到6.5万户、16.7万人，占非农业人口的9.2%，共发放低保金8513.4万元 ，基本实现了应保尽保。大力推进“福星工程”建设，全市五保老人集中供养率达到71 %。积极实施农村特困户救助，共向5.2万人发放救助资金723万元。切实维护群众合法权益，全 市共清收拖欠工程款1.87亿元，偿还农民工工资2548万元。严肃查处了征用土地中侵害农民利益、城镇拆迁中侵害居民利益、企业改制重组和破产中侵害职工利益等突出问题，以及教育、医疗乱收费等行为。大力实施扶贫济困工程，全市绝对贫困人口减少10万人，温饱巩固 率达到92%。人民生活水平继续得到提高，全市城镇居民人均可支配收入达到5864元，农民 人均纯收入达到2485元，城乡居民人均储蓄存款达到3540元，分别比上年增长6.6%、12.8%和21.6%。

　　——统筹经济社会发展，促进社会全面进步。坚持把教育放在优先发展位置，城乡义 务教育 收费全面实行“一费制”，全市完成危房改造21万平方米，小学学龄儿童入学率达到99.9%。以黄冈师院为龙头的高等教育、以黄冈职院和鄂东职院为龙头的职业教育、以黄冈中学为龙头的基础教育竞相发展。科技创新能力增强，科技特派员试点工作取得实效，科技进步对经济增长的贡献率继续提高。加强城乡医疗卫生基础设施建设，公共卫生体系进一步健全。及时采取有效措施，夺取了阻击高致病性禽流感的阶段性胜利。群众性体育活动蓬勃开展，竞技体育取得新成绩。高度重视人口、资源、环境工作，人口自然增长率控制在省下达的计 划内，关爱女孩、打击“两非”工作经验在全国人口和计划生育工作大会上交流;加强了对土地、水、森林、矿产等资源的节约保护与合理利用，环境保护和生态建设力度加大。加强 爱国主义、集体主义、社会主义和公民道德教育，深入开展精神文明创建活动，公民素质不断提高。积极促进文化事业和文化产业发展，社会科学、文学艺术、新闻出版、广播影视等 事业繁荣活跃，涌现了一批优秀精神产品。民族、宗教、对台、外事、侨务、人防、档案 、方志、保密、信访、老龄、残疾人等各项工作都取得新成绩。

　　——加强社会主义政治文明建设，大力建设法治政府。自觉接受人大依法监督和政协民主监督，广泛征求和听取人大代表、政协委员对政府工作的意见，人大代表建议和政协委员提案的办结率和满意率进一步提高。健全民主制度，推进政务、村务、厂务公开，较好地保证了 人民群众依法直接行使民主权利。认真贯彻实施《行政许可法》和国务院《全面推进依法行政实施纲要》，制定和实施了市政府《关于全面推进依法行政的若干意见》。积极开展国防教育和“双拥”活动，支持部队建设，做好军转干部、复员退伍军人接收安置和优抚工作， 军政、军民关系进一步密切。强化社会治安综合治理，深入开展“打造平安黄冈，创建安全 城市”活动，依法严厉打击各种犯罪和黑恶势力，严防和打击“法轮功”等邪教破坏活动， 重视人民内部矛盾纠纷排查调处和信访接待工作，集中处理信访突出问题及群体性事件，维护了社会稳定。全面落实安全生产责任制，强化重大危险源监控管理，安全生产和消防工作 受到省政府表彰。深入开展反腐败斗争，狠抓领导干部廉洁自律、查处大案要案和纠正行业 不正之风，廉政建设得到进一步加强。

　　各位代表!过去一年成绩来之不易。这是在党中央、国务院、省委、省政府和市委正确领导 下，全市人民团结拼搏的结果，是黄冈历任领导班子打下良好基础和市人大、市政协有效监督与关心支持的结果，是驻黄部队、武警部队、各民主党派、工商联、社会各界积极参与的 结果，是上级有关部门、外地黄冈籍人士、国内外各界朋友尤其是来黄冈投资的客商鼎力相 助的结果。在此，我代表市人民政府，向全市各条战线的广大干部群众，向所有关心、支持 我市经济社会发展的海内外人士，表示衷心的感谢并致以崇高的敬意!

　　各位代表，在总结成绩的同时，我们也清醒地看到，政府工作中还有不少薄弱环节，经济社会生活中还存在一些突出矛盾和困难。主要是：经济增长速度还不够快，虽然大部分预期目 标完成情况比较理想，但与全国、全省和周边地区竞相增长的态势仍有一定差距;经济增长的基础不牢，尤其是粮食增产、农民增收的长效保障机制不健全，工业支柱产业和骨干企业不突出，民营经济和外向型经济发展不够，投资和创业环境仍有待进一步优化;各级财政比较困难，地方可用财力和保障能力有限，收支矛盾突出;就业再就业压力大，社会保障覆盖面不广，部分下岗职工、城镇低收入居民和贫困地区群众的生活仍比较困难;政府自身建设 特别是行政能力建设、勤政廉政建设，以及抓落实的工作，还需进一步加强。对这些困难和问题，我们一定高度重视，积极采取措施，努力加以解决。

　　二、2005年工作安排

　　今年是全面实现“十五”目标、衔接“十一五”发展的重要一年。新一年政府工作总的指导 思想是：以邓小平理论和“三个代表”重要思想为指导，认真贯彻党的十六大、十 六届三中 、四中全会、中央和全省经济工作会议精神，全面落实科学发展观，牢牢把握国家加强和改善宏观调控、“促进中部地区崛起”以及建设武汉城市圈的战略机遇，继续坚持“全党抓经济，重点抓工业，突出抓招商，着力抓环境”的工作思路，进一步加大改革开放、结构调整 和转变经济增长方式的力度，全面实施三个百亿投入工程、农业五带十区工程、扶贫济困工程、文明创建工程、干部人才素质工程，着力解决经济社会发展中的突出矛盾和关系群众切 身利益的突出问题，确保农民增收、企业增效、财政增长、就业增加和社会稳定，加快赶超 全省发展水平和构建和谐黄冈步伐。全市经济社会发展主要预期目标是：生产总值增 长10 %，全社会固定资产投资增长20%，社会消费品零售总额增长11%，吸收外商投资增长20%，外贸出口增长15%，地方一般预算收入增长10%以上，城镇居民人均可支配收入增加300元，农民人均纯收入增加200元，居民消费价格涨幅控制在4%以内，城镇新增就业3万人 ，城镇登记失业率控制在4.4%以内，人口自然增长率控制在6‰以内。

　　确定上述目标，充分考虑了各方面的条件和需要，一是有较有利的宏观环境。世界经济保持较快增长，贸易扩大，投资活跃，有利于我们利用国外市场和资源;国家加强和改善宏观调控，实施稳健的财政货币政策，加大对农业等薄弱环节的扶持，加大对中部地区和贫困地区的扶持力度，我省大力推进县域经济发展和武汉城市圈建设，以及沿海产业加快向中西部转移，为黄冈加速发展提供了重大战略机遇。二是有较有力的增长支撑。近年来我们大抓招商引资，大搞项目建设，大力推进新型工业化和农业产业化，培植了一大批新的经济增长点。 同时，农业政策拉动效应继续增强，企业民营化和市场化机制不断完善，民间投资活力将随 着投资体制改革的深入而得到激发，都为今年经济加快发展提供了有利条件。三是有较强烈的内在要求。解决当前经济社会生活中的各种矛盾和问题，实现全面建设小康社会和赶超全省发展水平的宏伟目标，要求我们必须以积极进取、加快发展的指导思想来安排部署工作。

　　完成今年政府工作的目标任务，必须抓好以下十个方面的工作：

　　(一)以农民增收为核心，加快农业和农村经济发展

　　坚持把解决好“三农”问题作为经济工作的重中之重 ，坚决贯彻“多予少取放活”的方针，以农民增收为核心，以结构调整为主线，以提高农业综合生产能力为重点，加快农业和农村经济发展。

　　切实提高农业综合生产能力。坚决实行最严格的耕地保护制度，确保基本农田总量不减少、质量不下降、用途不改变。切实做好完善农村土地二轮延包工作，尊重和保障农户拥有承包地和从事农业生产的权利，尊重和保障外出务工农民的土地承包权和经营自主权。抓好优质粮产业工程和高产农田建设，稳定发展粮食生产。抓好“沃土工程”，努力培肥地力。立足抗灾夺丰收，加强农田水利和生态建设，提高农业抗御自然灾害的能力。

　　加快推进农业和农村经济结构调整。进一步抓好“五带十区”工程建设，盘大特色产业，扶持壮大龙头企业，推进龙头企业和基地、农户联结，增强龙头企业的辐射力和带动力。加快 实施“无公害食品行动计划”，大力发展优质无公害农产品，争创无公害、绿色和有机农产品标识。

　　加大科技兴农力度。突出抓好优质高产良种应用、高产高效模式、轻型简化栽培、无公害农产品标准化生产、高产优质配套养殖、动植物疫病防治及避灾技术等实用技术的推广应用。积极探索新形势下农技推广体系建设的新路子，按照公益性职能与经营性服务分开的原则，构建精干高效的公益性服务机构，加快小型适用先进农机具的应用，加强农业科技示范和指导，提高农业社会化服务水平。

　　继续发展“打工经济”。实施“阳光工程”，加强农民培训和职业教育，提高农村劳 动力素质，搜集掌握企业用工信息，有组织地推进农村劳务输出。

　　深入推进扶贫开发。继续实施整村推进，积极开展产业扶贫，加强对口支援和帮扶， 加大扶贫资金投入、项目建设和科教培训力度，努力改善贫困地区生产生活条件。

　　(二)坚定不移地重点抓工业，全力推进新型工业化

　　继续把发展工业作为黄冈立市、兴市、强市的根本大计，坚持以提高工业经济运行质量和效 益为中心，以培育有竞争力的产业为关键，以项目为载体，加快实施新型工业化规划。

　　集中力量培育有竞争力的产业和新的工业增长点。进一步加大工业技改投资和招商引资力度，着力发展和改造提升食品饮料、医药化工、纺织服装、建筑材料、机械电子五大重点产业 ，充实整合资产存量，培植一批利税过千万元、过亿元的工业小巨人，发展一批“专、精、特、新”的中小企业，形成一批具有地方特色和核心竞争力的工业骨干企业和农业产业化龙 头企业。加强经济运行监测与调节，着力缓解煤电油运等瓶颈制约，努力保障重点企业生产 和居民生活需要。

　　继续加强开发区和工业园区建设。优化产业定位，整合资源，主攻强项，形成特色， 着力建设一批服务优良、管理规范、功能完善、产业配套、优势明显的开发区和工业园区。采取得 力措施，优化配套服务，千方百计吸引外地客商到园区投资办厂，鼓励本地民间资本向园区 集中创业，以园区为依托打造产业集群。

　　加大对经济发展尤其是工业的信贷投入。积极支持金融机构调整优化信贷结构，加大 对重点 行业、企业、产品和项目的资金支持。加强信用担保体系建设，加大对特定领域、行业和群体的支持力度，完善“小额农贷”，推行“大额农贷”，增加中小企业尤其是民营企业流动 资金贷款，扩大对下岗失业人员小额担保贷款，支持县域经济发展。支持金融机构做好不良贷款清收和处置工作，降低不良资产比重，提高信贷资产质量。进一步强化信用环境建设，营造和维护良好的经济金融环境。

　　大力实施品牌战略和质量振兴战略。加大资源整合力度，积极推动资本、技术等要素 向优势企业和优势产品聚集，支持企业提高持久性开发和自主创新能力，着力打造知名品牌，引进国内外著名品牌，争创驰名商标和全省、全国名牌。

　　加快建筑业发展步伐。大力发展工业建筑和民用建筑，加大对建筑企业的改革脱困力 度，做大做强骨干建筑企业，扩大市场份额，争创建筑业强市。

　　(三)毫不动摇地突出抓招商，全面推进对外开放

　　着力提高招商引资工作水平。进一步解放思想，努力扩大招商引资规模，提高利用外资质量 。创新思路，强化措施，推动招商引资工作由遍地开花向招商选资、由招管一体向招管分离 、由各自为战向园区集中、由主要依赖政策效应向主要发挥环境优势、由以奖为主向责任考核转变，重点推行园区整体招商、专业小分队招商、以商引商、产业链招商等招商形式，围绕主导产业，积极引进国外市外战略投资者和大公司、大财团，推动配套项目投资、行业产业链延伸和企业群扩充。抢抓“中国光彩事业大别山黄冈老区行”的机遇，努力使一批大公司、大项目落户黄冈。

　　努力扩大外贸出口。认真落实国家和省鼓励出口的一系列政策措施，继续推行出口市场多元化战略，鼓励民营企业、高科技企业扩大出口，着力转变对外贸易增长方式，优化出口商品 结构，提高我市经济的外贸依存度。加快医药化工产品出口基地建设步伐，形成外贸、外资 、外经的联动效应。加强与海关、口岸、商检等部门的联系与沟通，改善进出口条件。扩大对外经济技术交流与合作，带动技术、设备、原材料出口和劳务输出。

　　积极融入武汉城市圈。坚持“市场主导、政府引导、企业主体、产业集群”的原则，积极推进与武汉以及圈内城市在企业改组联合、产业链延伸、产品开发等方面的协作，加快启 动一批能够促进产业形成和发展的重大项目。积极推进区域市场一体化，包括资本市场、流通市场、科技人才等要素市场资源的整合对接，在区域经济的交流融合中发展自身。

　　(四)进一步深化改革，激发经济社会发展活力

　　深化农村各项改革。认真抓好取消农业税、对种粮农民实行直接补贴、良种补贴和农 机具购置补贴等支持粮食生产的各项政策落实，继续深入推进农村税费改革、乡镇综合配套改革和村级债务化解工作，强化涉农收费专项治理，严格规范农村公益事业建设“一事一议”制度 ，确保农民负担不反弹。认真抓好农村信用社改革试点，改善农村金融服务。继续抓好粮食流通体制、国有农场管理体制、农村科技推广体制、卫生体制、血防体制、动物疫病防治体 制等各项改革。

　　深化企业改革。继续坚持以产权制度改革为核心，以民营化为取向，落实责任，加快 进度，全面完成国有、集体企业改革任务，实现市、县、乡、村所属公有制企业全面改制为民营企业 。按照建立现代企业制度的要求，健全公司法人治理结构，推进企业转换经营机制。加强国有资产监管，依法履行出资人职责，确保国有资产保值增值。进一步抓好市政公用事业、交通、水利等建管体制和科技、教育、文化、卫生等社会事业领域的改革，抓紧落实方案，积极稳步推进，尽快完成生产经营性事业单位转企改革任务。

　　深化财政管理体制改革。全面推行比较规范的部门预算，建立财税库横向联网制，实 行“阳光编制”和“阳光财政”。进一步完善政府采购操作规程和运行机制，将政府采购融入部门预算、集中收付和收支两条线管理改革之中。认真落实收支两条线管理制度，结合非税收入收支管理制度改革，将行政事业性收费、政府性基金、罚没收入等全部纳入财政预算管理或 专户管理。深化财政支出管理制度改革，严格预算管理，硬化预算约束，优化支出结构，降低财政供养系数，抓紧建立农村义务教育和县级政法工作最低经费保障制度，确保法定支出和重点支出。

　　(五)着力发展市区经济和县域经济，促进区域协调发展下大力发展市区经济尤其是市级经济。适应行政尤其是财政管理体制改革不断深化和 区域经 济竞相发展带来的挑战，坚持培植产业和提高品位并重，发挥文化、教育、生态和政治优势 ，实施高强度投入，高起点建设，超常规发展，快速提升黄冈市区在区域经济社会发展中的 龙头地位。特别要紧紧抓住“产业兴市”这根主线，突出产业定位，大搞招商引资，大办工 业企业，大兴特色产业，进一步加快开发区特别是东西湖工业园区发展，尽力支持汇源果汁、太子奶、稳健工业园等重点项目建设，以产业支撑市区发展，丰富城市内涵。

　　以民营经济为主体加快发展县域经济。落实省扩大县级经济社会管理权限、扩大扩权县市试点范围等支持县域经济发展的政策措施，集中精力抓好县域工业发展，培植骨干财源，加快推进税收过千万元企业的建设;充分发挥工业园区的载体作用，以优势企业和优势产品为依托，实施产业聚集和产业配套，发展具有地方特色的板块经济，提升县域工业化水平。坚持城乡并举和县市域经济民营化的取向，着力消除制约民营经济发展的观念、政策和体制性 障碍，加大政策扶持力度、依法保护力度和经济环境综合整治力度，支持民间资本进入法律法规未禁止的领域，努力形成一批有影响、有带动力的民营企业和企业集团。

　　加速推进城乡一体化进程。高标准抓好城镇规划编修，进一步强化城镇规划管理。以市县城区和中心镇、口子镇为重点，加快城镇基础设施配套建设，增强城镇整体功能。继续完善“ 门前四包”等长效管理机制，加大洁绿亮美力度，提高城镇管理水平，争创省级园林城市。 突破限制农民进城的体制性和政策性障碍，加大服务和维权力度，促进城乡人财物自由流动 ，以城带乡，共同繁荣。

　　(六)努力扩大投资和消费需求，培植新的经济增长点

　　千方百计加强重点项目建设。结合招商引资突破性地加大工业项目投入，集中力量抓 好一批产业关联度和附加值高、对全市经济发展具有重大带动作用的工业项目。继续抓好基础产业 、基础设施和城乡公共事业项目建设。能源方面，加快大别山电厂、白莲河抽水蓄能电站和黄冈天然气工程建设，确保按期建成投产。全面完成县城电网改造工程，建成220千伏浠水 至崔家湾线，形成黄冈东部环网;交通方面，重点抓好江北一级公路全线贯通、武汉至红安 一级公路、阳枫线举水大桥、阿深高速、武英高速、武麻高速等重点工程开工建设，进一步 优化路网结构;农林水利方面，重点建设大型优质商品粮生产基地和名特优农产品商品基地、退耕还林、德援项目和日元贷款造林项目、原料林基地、农村人畜饮水解困、大中型水库 整险加固、重点泵站更新改造以及灌区节水改造等工程;公共卫生方面，进一步抓好市县两级疾病防控中心、紧急救援中心和市传染病医院、县市传染病区、乡镇发热门诊项目建设。 抢抓今年中央预算内资金向农业和农村发展、社会事业建设、生态建设、环境保护、医疗卫生、重大基础设施建设倾斜的机遇，积极做好重点项目的储备、筛选和申报工作，力争有更 多的重点项目进入国家和省计划“笼子”。

　　着力培植消费热点。抓好经济适用房建设，深化住房分配制度改革。大力发展物业管 理、家政服务、社区服务等新兴服务业。继续加快城乡路、电、水、广播电视、现代通信等基础设 施建设，大力推进“村村通”或“全覆盖”工程，改善居民消费环境。建立沟通小城镇和农村地区的连锁营销网络和配送系统，加强市场监管，打击假冒伪劣，突出抓好食品和药品安 全工作，促进居民扩大消费。加大创建中国优秀旅游城市的工作力度，力争今年国家验收达 标。抢抓国家实施红色旅游“121”工程的机遇，强化培育精品、系列开发观念，把发展旅 游产业与对外开放结合起来，加强区域合作，形成跨省市一体化旅游网络，努力使旅游业跃上新的台阶。

　　重视做好物价工作。健全价格监测体系和预警机制，严格控制出台政府提价项目。重点加强能源、重要农业生产资料和粮食、医药等商品价格调控工作，整顿和规范医疗、教育、通信 、中介、建设项目等价格和收费秩序，抓好行政事业性收费清理和规范管理，全面推行费价公示制，完善居民消费价格调整听证会制度。

　　(七)高度关注民生问题，切实维护人民群众切身利益

　　切实做好就业再就业工作。坚持把促进扩大就业放在经济社会发展更加突出的位置，逐步确立有利于扩大就业的经济结构和增长模式，加快发展就业容量大的第三产业和劳动密集型产 业，大力促进民营企业发展，千方百计增加就业机会。继续落实就业再就业的各项优惠政策 ，加强就业指导和服务，抓好就业培训，鼓励各类下岗失业人员通过各种方式实现灵活就业 再就业，坚决维护农民工合法权益，健全再就业援助制度，切实解决就业困难人群的就业问 题。

　　加强社会保障体系建设。继续抓好“两个确保”和城市“低保”工作，认真落实企业退休人员基本养老金和低保对象“低保”标准调整政策，确保国有企业下岗职工基本生活费和离退 休人员基本养老金按时足额发放，确保失业人员和“低保”对象按规定及时足额领到失业保险金和“低保金”。进一步扩大社会保险覆盖面，加大社会保险费的依法征收力度，实现参 保率、缴费率、征缴率“三个净增长”。以改组改制企业分流人员和中断缴费人员为重点， 抓好社会保险接续工作。按照“分级负责、分类处理”的原则，妥善解决好困难人员养老、医疗保险问题。高度重视解决农村贫困人口生活困难，及时有效做好农村特困救助工作。

　　努力为人民群众办实事。市政府承诺今年要着力办好八件实事：一是完成全市农村公 路建设 1000公里，其中通乡油路250公里，通村油路350公里，通村公路400公里;二是将愿意集中 供养的五保户全部纳入福利院集中供养，对自愿分散供养的按每人每年800元落实供养待遇 ，全面启动城乡困难群众大病医疗救助工作;三是基本完成全市中小学校24万平方米的D级 危房改造;四是完成黄州至罗家沟段公路改扩建工程;五是完成黄州大道南端扩宽及管网配 套工程建设;六是基本完成城市防洪工程，解决市区排涝问题;七是完成黄州开发区110千 伏变电站建设;八是基本解决市直原预算内国有破产工业企业退休人员医疗保险问题。

　　(八)加快实施科教兴市和人才强市战略，为经济社会发展提供人才保证和智力支持

　　推进科技与经济紧密结合。 着眼于降低资源和环境代价，转变经济增长方式，努力通过技术 创新来保持和促进经济快速增长。大力实施“科技致富工程”和“技术创新工程”，大力引 进新工艺、新技术、新设备、新材料，改造提升传统产业，发展高新技术产业。健全技术研 究和开发体系，支持企业技术中心建设，增强企业的研究开发能力。加大对生产力促进中心 和科技型中小企业的支持力度，促进特色工业发展。

　　优先发展教育事业。深化教育改革和调整，全面推进素质教育。健全农村义务教育投入保障 机制，巩固提高“普九”成果。大力发展职业教育，加强职业技能培训。加强教师队伍建设 ，改善办学条件，提高办学质量和水平。加强黄冈中学、黄冈师院、黄冈职院和鄂东职院等 重点院校建设。严格规范各类学校招生和收费制度，加强学校财务管理和监督，严格治理教 育乱收费。推进和规范社会力量办学，促进民办教育健康发展。进一步贯彻实施《中华人民共和国国家通用语言文字法》，增强语言文字规范意识。

　　加强人才队伍建设。树立“人才是第一资源”的观念，抓住人才培养、引进、使用三 个环节 ，打破常规，不唯学历，不唯资历，不唯身份，不唯职称，只唯能力和本领，在分配中打破 平均主义，在人才使用中打破论资排辈和以身份定人，积极为人才合理流动创造条件，让想干事的人有机会，能干事的人有舞台，干成事的人有地位。

　　(九)加强社会主义政治文明和精神文明建设，全面推进社会进步

　　加强思想道德建设。坚持用邓小平理论和“三个代表”重要思想武装广大干部和群众 ，加强 爱国主义、集体主义、社会主义教育，努力在全社会形成共同的理想信念和精神支柱。加强社会公德、职业道德、家庭美德和诚信教育，在全社会形成良好的基本道德规范。强化以德 育人，优化文化环境，完善工作机制，重点抓好未成年人思想道德建设和大学生思想政治教育。

　　加强民主法制建设。自觉接受人大监督，认真执行人大决议。加强同政协和各民主党派、工商联、无党派人士的民主协商，认真听取工、青、妇等人民团体和决策咨询研究机构的意见 ，推进民主科学决策。加强基层民主制度建设，完善公开办事制度。坚持依法治市，推进政 务公开，增强政府工作透明度，提高政府公信力。加强行政司法工作，确保法律法规的严格 实施。加强律师、公证等法律服务，做好法律援助工作，依法保障妇女、未成年人和残疾人 的合法权益。加强普法宣传教育，在全社会营造学法、懂法、守法的良好氛围。高度重视和 做好维护社会稳定工作。坚决防止和纠正损害群众合法权益的行为，逐步把维护群众切身利 益的工作纳入规范化、制度化、法制化轨道。认真开展经常性的矛盾纠纷排查调处工作，努 力把各种矛盾消除在萌芽状态。认真贯彻实施信访条例，建立以督查督办为重点的信访工作 机制，集中处理好信访突出问题及群体性事件。坚持“打防结合、预防为主”的方针，深入开展“创安”活动，强化治安基础防范工作，完善治安防控体系。依法严厉打击各种刑事犯 罪、经济犯罪和黑恶势力，提高大案要案侦破率。建立健全打击“法轮功”等邪教组织的长 效机制，抓好反邪教警示教育。坚决查禁黄赌毒等各种社会丑恶现象，净化社会环境。高度 重视和切实抓好安全生产，落实安全生产责任制和重大危险源监控制度，努力实现安全生产 形势的进一步好转。

　　认真贯彻“三大基本国策 ”。加强人口和计划生育工作。加强计划生育尤其是流 动人口、城市下岗、待岗和破产企业职工计划生育管理，稳定低生育水平。完善基层计划生育基础设 施和服务体系。加大综合治理力度，深入开展“关爱女孩”行动，逐步解决出生人口性别比 失衡问题。加强资源管理工作，依法保护和开发各类资源，重点抓好土地、水、森林、矿产 等资源的保护和合理开发利用。在严格土地管理的同时，走内涵挖潜之路，用好存量土地，盘活闲置土地，转化低效益土地，提高单位面积投资率和创税率。加强生态环境建设。继续 抓好退耕还林、湿地自然保护区等一批重点生态工程建设。重视环境保护，发展循环经济，推进清洁生产，严格控制污染物排放，构建资源节约型的产业结构和消费结构。

　　大力发展文化、卫生、体育等社会事业。加大对文化事业的支持力度，完善文化产业 政策，促进文化事业和文化产业共同发展。进一步发展文学艺术、新闻出版和广播影视业，重视发展哲学社会科学，实施精品工程，为人民群众提供更多的优秀精神产品。加强文化市场建设 与管理，整治和规范“网吧”经营，净化文化市场环境。继续加强公共卫生体系建设，抓好 血吸虫、艾滋病、结核病等重大传染病的防治。积极稳妥地推进新型农村合作医疗试点，逐步建立由政府组织引导，农民自愿参加，个人、集体和政府多方筹资，以大病统筹为主的农 民医疗互助共济制度。积极推动群众性体育活动蓬勃开展，抓好竞技体育，提高城乡居民健 康水平。

　　认真做好民族、宗教、对台、外事、侨务等工作。全面贯彻党的民族政策，依法加强 对宗教 活动的管理。提高对台工作水平，扩大利用台资。充分发挥外事、侨务等部门的纽带和桥梁作用，引进国外智力，进一步扩大对外交往与合作。大力发展残疾人事业。认真做好档案、方志、保密、老龄等工作。

　　积极支持部队和国防建设，巩固军政军民团结。关心和支持驻黄部队和武警部队建设 。抓好人民防空和民兵预备役工作。深入开展国防教育和“双拥”活动，健全国防动员体系，做好军队转业干部、复员退伍军人和离退休干部接收安置和优抚工作。

　　(十)加强政府自身建设，提高行政能力

　　加强行政能力建设。积极推进政府管理体制和机制创新，把财力物力等公共资源更多 地向社会管理和公共服务领域倾斜，提高运用经济和法律手段管理经济和社会事务的能力。深化县市机构改革和干部人事制度改革，切实降低行政成本，提高行政能力和效率，构建服务型政府。推进政企、政资、政事分开，解决好政府管理中缺位、错位、越位问题。着力提高因地 制宜、谋划发展的能力，抢抓机遇、加快发展的能力，市场运作、破解难题的能力，深化改 革、开拓创新的能力，统筹协调、和谐创业的能力，努力形成政府创新环境、部门创优服务 、企业创业发展、群众创造财富的局面。认真贯彻实施《行政许可法》，落实执法责任制、 执法过错追究制和行政赔偿制度，真正做到有权必有责、用权受监督、违法受追究、侵权要 赔偿、执法有保障。认真搞好经济普查工作，做好“十一五”规划和2020年远景目标的制定工作，把考虑长远发展、可持续发展与解决好当前突出矛盾和问题结合起来，把积极进取精神同科学求实态度结合起来，提高规划的预见性、科学性和指导性，多做打基础、埋伏笔、 作铺垫、管长远的事情。

　　加强政风建设。牢固树立正确的权力观和政绩观，坚持把群众是否满意、是否受益作 为衡量政绩的根本标准。大兴求真务实之风，坚决反对形式主义、作表面文章，坚决反对弄虚作假 、虚报浮夸，坚决取消不必要的检查评比，不搞脱离实际的升级达标。下大力精简会议、文件，改进会风、文风，减少应酬，深入基层、深入实际、深入群众调查研究，及时解决基层和群众关心的热点难点问题。坚决反对讲排场、比阔气、铺张浪费的恶劣风气，弘扬艰苦奋 斗的优良传统和作风，坚持艰苦创业、勤俭办一切事业。加强廉政建设和反腐败斗争，认真落实党风廉政建设工作责任制，严格遵守反腐倡廉的各项规定，从严防范、惩治公务人员失 职渎职行为和职务犯罪，建立和完善教育、制度、监督并重的预防和惩治腐败体系，从源头 上预防和消除腐败。在政府系统深入开展保持共产党员先进性教育活动，大力倡导克难奋进 的精神、奋勇争先的精神、勇于负责的精神、开拓创新的精神、苦干实干的精神，自觉做到 为民、务实、清廉。建立健全经济责任审计制度，对县处级以上领导干部全面实行经济责任 审计;加大大案要案查处力度，以卓有成效的反腐倡廉成果取信于民。

　　进一步加大工作落实力度。牢固树立“言必行、行必果”的诚信理念，围绕市委的决策部署 和人代会确定的任务，进一步健全政府工作责任制，完善目标责任管理考核体系和办法，严 格工作督查制度，敢于坚持原则，敢于触及矛盾，敢于批评错误，反对好人主义，反对敷衍塞责，反对推诿扯皮，特别要把评判工作实绩的权力真正交给公众，以群众和舆论的力量来及时跟踪督办工作落实，让人人起来负责，确保已经决定的工作事项一件一件落到实处。

　　各位代表!今年我市经济社会发展的任务十分繁重。我们一定要紧密团结在以胡锦涛同志为 总书记的党中央周围，在市委的领导下，紧紧依靠全市人民，解放思想，与时俱进，开拓创新，扎实工作，奋力赶超全省发展水平，为全面完成“十五”计划，加快黄冈振兴崛起而努力奋斗!
